# Missing Data Approaches in eHealth Research: Simulation Study and a Tutorial for Nonmathematically Inclined Researchers

**DOI:** 10.2196/jmir.1448

**Published:** 2010-12-19

**Authors:** Matthijs Blankers, Maarten W J Koeter, Gerard M Schippers

**Affiliations:** ^2^Amsterdam Institute for Addiction Research (AIAR)Academic Medical Center, University of AmsterdamDepartment of PsychiatryAmsterdamThe Netherlands; ^1^Arkin AcademyAmsterdamThe Netherlands

**Keywords:** Missing data, multiple imputation, Internet, methodology

## Abstract

**Background:**

Missing data is a common nuisance in eHealth research: it is hard to prevent and may invalidate research findings.

**Objective:**

In this paper several statistical approaches to data “missingness” are discussed and tested in a simulation study. Basic approaches (complete case analysis, mean imputation, and last observation carried forward) and advanced methods (expectation maximization, regression imputation, and multiple imputation) are included in this analysis, and strengths and weaknesses are discussed.

**Methods:**

The dataset used for the simulation was obtained from a prospective cohort study following participants in an online self-help program for problem drinkers. It contained 124 nonnormally distributed endpoints, that is, daily alcohol consumption counts of the study respondents. Missingness at random (MAR) was induced in a selected variable for 50% of the cases. Validity, reliability, and coverage of the estimates obtained using the different imputation methods were calculated by performing a bootstrapping simulation study.

**Results:**

In the performed simulation study, the use of multiple imputation techniques led to accurate results. Differences were found between the 4 tested multiple imputation programs: NORM, MICE, Amelia II, and SPSS MI. Among the tested approaches, Amelia II outperformed the others, led to the smallest deviation from the reference value (Cohen’s *d* = 0.06), and had the largest coverage percentage of the reference confidence interval (96%).

**Conclusions:**

The use of multiple imputation improves the validity of the results when analyzing datasets with missing observations. Some of the often-used approaches (LOCF, complete cases analysis) did not perform well, and, hence, we recommend not using these. Accumulating support for the analysis of multiple imputed datasets is seen in more recent versions of some of the widely used statistical software programs making the use of multiple imputation more readily available to less mathematically inclined researchers.

## Introduction

Missing data is a common nuisance in eHealth research [1,2]. Subjects may be unwilling or unable to respond to some items or may fail to complete sections of questionnaires due to lack of time and interest, thus leading to data “missingness.” In longitudinal studies, participants may drop out early or be unavailable during one or more data collection waves. If not addressed properly, data missingness can induce bias and corrupt external validity, which is both inevitable and uncontrolled by the researcher [3]. Because many of the statistical procedures used by researchers are designed to have complete datasets, it is important to handle missing data in a principled manner [4].

As dropout rates in eHealth studies tend to be relatively high and are even considered typical by some, addressing data missingness and dropout is of great importance. The observation that in any eHealth trial a substantial proportion of users drop out before completion has been called the “Law of Attrition” [1]. A recent review by Christensen and colleagues [2] provides an overview of dropout rates in eHealth interventions for depression and anxiety. Completion rates for online depression interventions ranged from 43% to 99%, with some trials indicating poorer retention after a longer follow-up. The results of one trial of an intervention to treat anxiety in this review reported a 6-month follow-up rate of 44% in the experimental group [2]. In reporting outcomes of a study with a considerable dropout rate, it is important to choose statistical techniques that are appropriate for the analysis of datasets with missing observations [5].

The primary concern when facing substantive missingness is that a study with high attrition rates may yield biased estimates (of the mean, for example) caused by a biased sample. Patients that leave studies prematurely have been shown to be more likely to be involved in drug use or deviant behavior [6-8], to have poorer academic performance, and to be less skillful in resisting peer pressure than other subjects [9]. Edlund and colleagues [10] found that sociodemographic characteristics associated with intervention dropout included low income, young age, and a lack of adequate health insurance coverage. Patient attitudes associated with dropout include viewing treatment as relatively ineffective and feeling embarrassed about seeing a mental health provider. Christensen and colleagues [2] identified several reasons for dropout from eHealth trials: time constraints, lack of motivation, technical or computer-access problems, a depressive episode or physical illness, the lack of face-to-face contact, preference for taking medication, perceived lack of treatment effectiveness, improvement in condition, and burden of the program. Therefore, dropout from eHealth interventions cannot be considered “random,” but may be based on participants’ characteristics, possibly leading to biased estimators if not addressed adequately.

In short, four key reasons for the use of missing data approaches should be recognized: (1) Missing data may compromise randomization integrity in randomized clinical trials, as drop-out rates may differ over the trial arms. (2) In all longitudinal study designs, missing data may introduce selection bias, as is made clear in the previous section. (3) An intention to treat analysis—as is requested in the consolidated standards of reporting trials (CONSORT) statement and in most other guidelines for the analysis of randomized (controlled) clinical trials (RCTs)—is a necessary step when clinical endpoints are missing for some of the participants [11]. (4) For all possible study designs, missing data may introduce a loss of power, some of which may be won back by using appropriate missing data approaches.

Remarkably, the problems encountered and the solutions implemented while solving missing data problems are rarely mentioned outside the statistical literature [11]. As resources or even a theoretical framework are sometimes lacking, researchers, methodologists, and software developers resort to editing the data to disguise an appearance of completeness. Unfortunately, ad hoc edits, or not handling missingness explicitly, and analyzing data using only complete cases may do more harm than good. These approaches could lead to results that are biased, lacking in power, and unreliable [12]. In the same vein, inappropriate use of missing data approaches will lead to biased results. This will be discussed in more detail for one of the tested approaches, although it applies to each of the other techniques as well. In general, in cases of data missingness, optimal analysis results will be obtained with the appropriate use of missing data approaches. Any other approach could lead to severe bias.

The aim of this paper is to provide a straightforward primer for eHealth researchers who seek solutions for missingness in datasets. To provide researchers with tools for working with data missingness, this paper reviews the strengths and weaknesses of the most common missing data approaches and tests the approaches in a simulation study. Theory on missingness patterns and the most widely used methods of handling missing data are comprehensively presented. The validity, reliability, and coverage of 9 different methods for dealing with incomplete datasets are presented. Some of these methods are relatively straightforward and basic, while others are more advanced and use computationally demanding algorithms to estimate missing values. Although the technical and mathematical details of the presented methods are outside the scope of this paper, those interested can consult with any of a number of references [12-16]. The primary goal of implementing any of the discussed approaches is to obtain unbiased estimators. This is achieved through the creation of datasets in which missing values are replaced by appropriate values to conserve the properties (ie, mean, variance, and distribution) of each variable. These imputed values together with the collected “real” data lead to unbiased estimates of parameters [12].

In general, 4 forms of missingness can occur in longitudinal studies: (1) In the case of initial nonresponse, no baseline data is collected for the participant, although follow-up measures may have been completed. (2) Loss to follow-up is the other way around: baseline data is collected, but (at a certain time point) the researchers fail to collect follow-up data. (3) Wave nonresponse is closely related to loss to follow-up in that data is not collected during one or more of the “waves,” but data are collected during earlier and later measurement waves. Missing data has to be interpolated if this form of missingness occurs. (4) The fourth form of missingness stems from item nonresponse. This occurs when a participant fails to respond to certain measures or questions, such as when some of the items from a questionnaire are skipped. For example, when the missing items are part of a highly correlated construct measurement (eg, one of the 16 items in a quality of life scale is missing), imputation is possible based on the other 15 collected item scores. In short, the selection of a missing data approach will in part depend on the form of missingness encountered. Although some of the presented methods may be efficacious at handling data problems, the most important determinant for preventing missing data values is to retain subjects in the study [17]. However, it often may not be feasible to invest extensive amounts of effort, time, and money to obtain nearly perfect response rates. Even then, small amounts of missing data may lead to substantial bias, depending on the pattern of data missingness.

### Patterns of Data Missingness

In general, 3 mechanisms of missingness are discerned: missing at random (MAR), missing completely at random (MCAR), and missing not at random (MNAR) [13]. Each of these 3 patterns can have its own implications for the effects of missingness on parameter estimates derived from the dataset. Although these 3 terms have formal statistical definitions, their practical meaning for the purpose of this paper is best described through examples [4].

Commonly, the probability that an observation is missing depends at least in part on information that is present: missingness is dependent on observed characteristics. This type of missing data generally is referred to as missingness at random or MAR [12]. The word “random” in MAR means something rather different from what most researchers typically think of as random. The randomness in MAR missingness means that once all data have been controlled for, any remaining missingness is random [4]. As long as missingness depends on available data, but not on unavailable (missing) data, the missingness pattern is considered MAR [12]. MAR can, for example, arise when an investigator studies the predictive validity of treatment adherence on the outcome of an intervention. If patients who drop out of treatment have a propensity for missing follow-up measurements, missing follow-up data may have an MAR missingness pattern. Missingness is dependent on a subjects’ characteristic (treatment adherence) that is available in the dataset.

Missing completely at random (MCAR) is a special case of MAR [12]. If cases with missing data form a truly random subset of the dataset, missing observations are considered MCAR. In essence, this means that correct parameter estimates (but not confidence intervals) can be obtained by using only the complete cases from the dataset. Typically, MCAR arises when a portion of questionnaire data from a study subject is accidentally lost. Missingness is completely random and the probability that an observation is missing is not related to any of the subjects’ characteristics. Sometimes, this missing data pattern is referred to as ignorable missingness [4].

If the probability that an observation is missing depends on an unmeasured factor, this factor is partly missing itself and therefore not available, or the value of the observation predicts its own probability for missingness, the missing data pattern is called missing not at random or MNAR [12]. MNAR can be referred to as nonignorable missingness. Estimators derived from a dataset with an unaddressed MNAR missingness pattern can be biased [4]. For example, asking a subject for his or her income level without collecting data related to income may lead to forms of missingness in correspondence with this MNAR pattern. People with high incomes may be reluctant to provide information on their earnings, so it might well be that missing data are more likely to occur when the income level is relatively high. Here, the predictor for missingness is related to unobserved characteristics of the subject. Because this predictor is not measured, imputing this missing value properly is complicated; for example, one would have to specify a distribution for the missingness [12].

In general, there is no way to test whether MAR or MNAR holds in a dataset [12]. More specifically, Graham [4] indicates that pure MCAR, MAR, or MNAR really never exists: these concepts require almost untenable assumptions. In reality, often a mixture of forms will be found. Collins et al (in [12]) demonstrated that in most realistic cases, an assumption of MAR where MNAR is at hand leads to only minor impacts on estimates and standard errors. MNAR missing data approaches require the analyst to make assumptions about the model of missingness; if this assumed model is incorrect, its results are unpredictable and probably biased. Because of difficulties in the straightforward application, MNAR methods are not widely used. In this paper, we therefore do not focus on missing data approaches for MNAR patterns.

For MAR and MNAR, it should be recognized that patterns of missingness and the consequences for derived estimators are not solely a characteristic of the data, but a combination of the available data and the planned analysis. For example, if an MNAR pattern in which an unobserved or unmeasured variable is predictive of missingness (for example, left or right handedness) but is not correlated with the endpoint of the study, then the MNAR pattern does not lead to biased estimators (only to a loss of power). Another example is pointed out by Graham [4]. Suppose one develops a smoking prevention intervention. Smoking in this example is measured at two time points: before the start of the intervention (t1) and one year later (t2). Suppose missingness at t2 is dependent on t1. If an analysis or missing data approach is performed under a model in which t1 is included, missingness on t2 follows an MAR pattern, whereas t2 would follow an MNAR pattern if t1 was not included. In other words, a biased estimator as a result of missingness can only occur in reference to a specific dependent variable under a specific statistical model. Some of the more advanced missing data approaches discussed in this paper use this characteristic to estimate and impute the missing values.

### Missing Data Approaches

Over the last couple of decades, several methods for handling missingness have been developed. In this section, a number of these missing data approaches are presented. The approaches that are most useful and applied most often are described below [4]. The first three approaches in this overview are considered “basic” as they are conceptually straightforward and require minimal computations, such as complete case analysis, listwise mean imputation, and last observation carried forward (LOCF). The “advanced” approaches are newer, require more computational power, and are conceptually more complex than basic approaches. Two of these advanced approaches are imputation techniques that replace missing values in the dataset with a single approximation; these approaches are regression imputation and expectation maximization imputation. The final four approaches are multiple imputation techniques replacing a single missing observation with multiple simulated values: NORM, MICE, SPSS MI, and Amelia II. The use of these last four approaches leads to multiple instances of the original dataset with a variance in the imputed values for the missing observations that resembles the accuracy (or inaccuracy) of the missing values approximation. See also Table 1.

**Table 1:** Missing data approaches in this study

Approach	Description	Missingness Pattern	Type
Complete cases	Only cases without missing observations in analysis	MCAR^a^	Basic, single
Mean imputation	Imputes missing observations with listwise mean for each variable	MCAR^b^	Basic, single
LOCF	Imputes the last available observation in the current data collection wave	-	Basic, single
Regression imputation	Imputes missing observations by prediction based on other variables in a regression model	MAR, MCAR	Advanced, single
EM imputation	Imputes missing observations using expectation maximization algorithm	MAR, MCAR	Advanced, single
NORM	Multiple imputes missing observations under a normal model	MAR, MCAR	Advanced, multiple
MICE	Multiple imputes missing observations using chained equations	MAR, MCAR	Advanced, multiple
SPSS MI	Multiple imputes missing observations under a normal model in SPSS	MAR, MCAR	Advanced, multiple
Amelia II	Multiple imputes missing observations using a bootstrapping-based algorithm	MAR, MCAR	Advanced, multiple

^a^ This approach will lead to unbiased point estimators (eg, means) under MCAR, but will result in lowered power and sample size.

^b^ This approach will lead to unbiased point estimators (eg, means) under MCAR, but will result in biased, smaller confidence intervals.

### Complete Case Analysis

The most popular and most often used missing data handling method is complete case analysis (casewise deletion). In complete case analysis, all cases with missing values are removed from the dataset before analysis. This method is straightforward in its application. This technique assumes MCAR and its application will lead to biased results under other patterns of missingness. Even under a valid assumption of MCAR data, this method is not preferential because the reduced number of cases used for the analysis leads to loss of statistical power [4].

### Listwise Mean Imputation

Listwise mean imputation, in which missing values of each variable are imputed with the arithmetic mean of the available observations for the variable, attempts to overcome the loss of power of complete case analysis. Like complete case analysis, listwise mean imputation assumes the MCAR missingness pattern, which is uncommon in empirical datasets with missing observations. If the data missingness pattern is not MCAR, imputing missing values with the listwise mean will result in a biased estimation of the mean. Under all missing data patterns (also MCAR), listwise mean imputation will reduce the variance of the variable. Imputed values equal to the mean do not contribute to the total variance. This leads to decreased standard errors and artificially small confidence intervals. Because of the inadequacy of listwise mean imputation to conserve the imputed variables variance, this method is considered by some to be one of the worst missing data approaches [18].

### Last Observation Carried Forward

The third most-often used method is last observation carried forward (LOCF). This approach is regularly used in epidemiological research, especially in clinical trials [19]. LOCF takes into account the individual’s previous observed value on a given variable [20]. If an observation at a certain data collection wave is missing, the last observed value is then used as an estimate for this missing observation. A related method, last observation carried backward (LOCB), works according to the same approach, but imputes a newer observation in the case of a missing earlier observation of the same individual. Both carried observation methods can only be used in longitudinal research designs with at least one complete observation. Despite its wide application in clinical trials, however, recent empirical studies have cautioned against the use of this technique [21] and have demonstrated its bias [22]. This bias mainly stems from the fact that imputing previously measured values can be conservative in some situations, but not in others. LOCF assumes there will be no further improvement and, therefore, underestimates the treatment effects in an effective intervention if the intervention’s effect is to change a current state (of well-being, for example). However, if the intervention’s expected effect is to slow down a decline (for example in a cognitive enhancement intervention for patients with Alzheimer’s disease), carrying forward a previous observation will exaggerate the found treatment effects. In RCTs, LOCF may also have unexpected anticonservative effects. In the control or placebo arm of a study, LOCF assumes no (spontaneous) change, which is not conservative because study participants in the control arm may improve as well. When there is an assumption of no change in the control condition, but in reality there is a change, larger differences between treatment and control arms in RCTs may be artificially produced. Suddenly, LOCF is not conservative anymore [23]. In general, in studies with relatively favorable baseline measures, LOCF will project these favorable baseline scores to clinical endpoints, thus exaggerating the efficacy of the intervention. Because of these unexpected anticonservative effects, we strongly advise against the use of LOCF.

### Regression Imputation

Regression imputation is the first of two “advanced” single imputation methods discussed in this paper. By adding randomly sampled “noise” from a normal distribution to a prediction model based on linear regression, the regression method imputes missing values based on the relations between variables in the dataset while preserving the variables’ variance. There is some discussion about the number of predictors that should be included in the model. In general, the use of more predictor variables in the regression equation is not necessarily better. A more parsimonious model, where only statistically significant predictors are retained, is usually a better model. However, it is important to keep in mind that two types of predictor variables should be retained in the model: those predicting the variable(s) with missing observations and those that predict missingness. The latter group of predictors help to correct for differential dropout-inducing bias to the estimators. In theory, regression imputation is applicable under both MCAR and MAR missingness patterns.

### Expectation Maximization Imputation

The other advanced single imputation method discussed here is based on expectation maximization (EM). The EM approach is a procedure that estimates unmeasured data and is based on iterating through two alternating steps [24]. In the expectation step, an appropriate value is calculated for the missing observation based on the available data and its distribution. In the maximization step, an appropriate value is calculated based on the current updated dataset. The model can be improved because original data will be used in addition to the proposed missing data imputations calculated during the most recent expectation step. These two steps are alternated numerous times: after each expectation step a maximization step will follow. After each iteration, a better model can be specified, leading to more accurate missing value estimations. After the final iteration, theoretically the most accurate estimation of the missing values is reached: the EM procedure will impute this value into the dataset as a replacement for the missing observation.

### Multiple Imputation

In recent years, multiple imputation (MI) has emerged as a methodology for handling missing data. Originally, it was viewed as being most appropriate for complex surveys, although in the 1990s it was shown to be valuable in other settings as well [14]. Multiple imputation is an approach in which the missing values are replaced by multiple simulated versions. “Multiple” refers to the custom of replacing missing values with several different values, typically between 3 and 10 [25]. Rubin [13] has shown that unless the rate of missing information is very high, there is simply little advantage to producing and analyzing more than 10 imputed datasets. Each of these “replacement values” can be estimated using regression equations, a form of EM, the identification of a “near-neighbour” donor case with matching properties, or through a combination of these methods. In any of these methods, the correlations between the different variables in the dataset are taken into account. Based on these correlations and other variable properties, appropriate estimations for the missing values are generated.

Missing values that are replaced with more than one possible estimator will produce more than one completed dataset: each of the 3 to 10 imputations leads to a new dataset containing the original “complete” available observations and the new “generated” imputed ones. Each of the 3 to 10 datasets is first analyzed as if it were a complete dataset with no missing values. The separate results can then be combined into one final result according to specific rules. Rubin [13] presented formulae to combine the estimators and standard errors obtained from the 3 to 10 imputed datasets into one estimator and one standard error. The combined estimator is the arithmetic mean of the 3 to 10 estimators obtained from the imputed datasets; the combined standard error is based on both the standard errors and the variance of the 3 to 10 estimators of the imputed datasets. The combined estimator and standard error can be used for the calculation of, for example, *t* test statistics and analysis of variance. A more recent paper shows how a variety of other test statistics can be calculated as well [26].

From a researcher’s perspective, the biggest advantage of MI is flexibility. It applies to a wide range of missing data situations and is simple enough to be used by nonstatisticians. Theoretically, this approach is superior to other models because it often produces the most robust effects. In this paper, four multiple imputation programs are compared. The first, called NORM [15], was developed for use under S-PLUS (TIBCO Spotfire, Somerville, MA, USA) or the R Statistical Programming Environment, but is also available as a stand-alone program. Using the NORM, one can perform multiple imputations of multivariate continuous data under a normal model. More information on its exact routines is presented in [15]. The second MI program is called Multivariate Imputation by Chained Equations, MICE [27], and was developed for use under S-PLUS, R, Stata (StataCorp LP, College Station, TX, USA), and as a stand-alone Windows program. MICE is an attempt to combine the most attractive aspects of MI approaches developed by [15] and [28]. The third MI program has been included in SPSS Statistics (SPSS Inc, Chicago, IL, USA) since version 17. According to the product information, this MI module allows for quick and accurate data estimates in cases where observations are missing [29]. The fourth MI program is called Amelia II, developed by Honaker and colleagues [30]. Amelia II multiply imputes missing data in a single cross-sectional dataset from time series data or from a time series cross-sectional dataset. This new bootstrapping-based algorithm it is presumed to be faster and more flexible than the other programs.

## Methods

### Source Data

The dataset in this simulation study was obtained from an online, self-help prospective study for problematic alcohol consumers. The online self-help program was developed by a substance abuse treatment center in Amsterdam, the Netherlands. Each new participant was invited for a measurement of alcohol consumption, quality of life, self-efficacy, and demographics. Data were collected at two waves, at baseline, and 3 months after baseline. All the cases with missing values were removed from the original dataset, resulting in a dataset with 124 cases, with 0% missing data. The dataset contains self-reported daily alcohol consumption quantities measured in standard drinking units containing 10 grams of ethanol. These consumption quantities were available at baseline and at the 3-months follow-up. For the purposes of this paper, we used only the subscale measuring alcohol consumption for the last 7 days, measured using Timeline Follow-Back methodology [31]. Currently, a randomized clinical trial (Netherlands Trial Register NTR-TC1155) on the effectiveness and cost-effectiveness of this intervention is in the process of being executed [32].

This complete (0% missing) dataset was used as a reference value for comparison of each approach. Next, one of the weekdays from the follow-up measurement was selected and an MAR missingness pattern was induced, leading to 50% MAR missingness in this variable. The operationalization of MAR applied by the execution of this macro is according to the method suggested by Scheffer [25]. Briefly, for this method two variables are necessary: (1) a variable predicting missingness and (2) a variable in which missingness will be induced. If the score of the missingness predictor variable is high, the chance that missingness will occur in the missingness variable is high; if the score of the missingnes predictor variable is low, the chance on missingness is also low. As a result, the proportion missing data in the missingness variable is correlated with the value of the missingness predictor variable.

After MAR induction, the missing data approaches were performed on the dataset with missing observations. For LOCF, data collected at baseline were carried forward to the missing follow-up measurement for the variable upon which missingness was induced. All “advanced” missing data approaches came with default software settings. It is possible to adjust these settings to change the number of iteration steps, convergence criteria, and the distribution of random error. For the presented analysis, the default software settings were used. To test sensitivity of the results to changes in these default software settings, the study was replicated using stricter, more calculation-intensive settings, that is, a larger number of iterations or stricter convergence criteria. The results obtained with these stricter settings did not differ systematically from the results obtained using the default settings.

To investigate reliability and coverage of the results obtained through these approaches, a resampling approach was performed. A total of 75 samples of n = 124 were drawn with replacement from the MAR imposed dataset, and these resampled datasets had, on average, 50% MAR missingness on the selected variable. Next, missing values from each dataset were imputed using the different approaches. Figure 2shows the arithmetic mean for the variable with imputed missing values. Each point represents the mean value of postintervention drinks, obtained from one of the 75 datasets. The area between the two dashed horizontal lines indicates the 95% confidence interval of the reference variable, which is the same variable indicating postintervention drinks but before MAR missingness is imposed. The white dot in each column indicates the mean for the repeated application of each missing data method on the 75 datasets.

Superior performance of the MI approaches over the other advanced approaches (and of the advanced approaches over the basic approaches) was expected, based on previous studies [12,25]. However, in contrast to previous publications, the approaches tested in the current study used a dataset with nonnormally distributed count data in order to mimic the everyday application of these approaches. Count data distributions are known for their deviation from the normal distribution [33].

### Validity, Reliability, and Coverage

For successful application in a variety of missing data situations, it is important to test for reliability in addition to validity. For example, will the use of the presented methods lead to comparable results with repeated application? Coverage can be regarded as a combined indicator for validity and reliability. It is expected that coverage of the advanced approaches will outperform the basic methods.

Validity was operationalized as the extent to which the estimate obtained by a missing data approach approximated the reference value. Validity (ie, test validity) was assessed by calculating *t* values and effect sizes for the differences between the reference value (mean variable score before MAR induction) and the imputed variable (mean variable score after the induction of MAR). Reliability was operationalized as the variance of the estimates obtained through repeated application of each missing data approach: the lower this variance, the smaller the confidence interval and the higher the method’s reliability. The third statistic calculated in this study was the coverage. This coverage statistic was calculated using the data obtained in the simulation study. It indicates the proportion of means within the 95% confidence interval of the mean reference value: when 70 of the 75 bootstrapped mean values for a missing data approach are within the 95% confidence interval of the reference value, the coverage is 70/75 or 93%. This coverage measure was previously used for comparable purposes, for example by Schafer and Graham [12].

## Results

**Figure 1:**
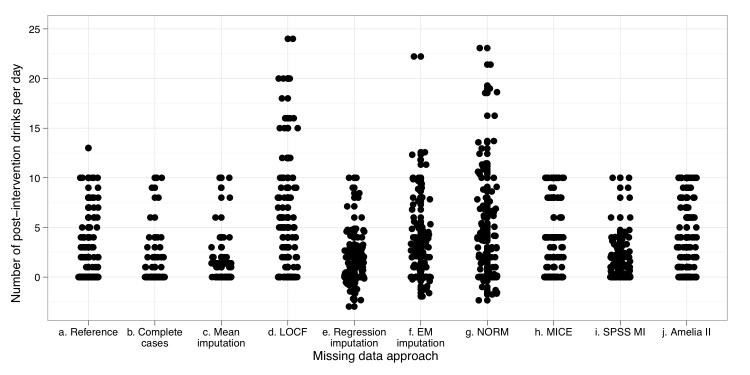
Strip chart for 9 missing data approaches and the reference value

The complete (reference) dataset and the datasets that resulted after application of the missing data approaches are plotted in Figure 1. Each point in this figure represents a single observation (number of postintervention self-reported drinks per day for each participant). The observations for the reference are shown in the first column (a) on the x-axis. These observations are the “true” complete observations before missingness was induced. Also shown on the x-axis (columns b to j) are the observations that were produced after the MAR 50% missingness was imposed and corrected by each of the nine missing data approaches. Some horizontal jitter was added to the strip chart to prevent equally valued observations from overlapping on the y-axis. Please note that plotting the ideal missing data approach’s observations would lead to results identical to the reference plot in Figure 1. On the y-axis, the number of postintervention drinks per day is indicated. For the 4 multiple imputation approaches (columns g to j), only the first created dataset was plotted.

A number of participants reported zero postintervention drinks per day; subsequently, their data points are plotted very close to each other. As is often the case for count data, observations are positive integers only and the distribution of the observations is nonnormal. Table 2 shows descriptive summary statistics for each of the methods compared with the reference. The application of complete case analysis, listwise mean imputation, regression imputation, and SPSS 17 multiple imputation led to an underestimation of the mean number of postintervention drinks. LOCF, and to a lesser extent EM imputation and NORM, led to an overestimation of the mean. Regression imputation, EM imputation, and NORM impute some negative values, which is impossible from an empirical point of view; however, these approaches could still produce unbiased estimators of mean and variance, for example. Applications of MICE and Amelia II produced the closest approximations to the reference mean value, as determined by visual analysis of the data.

**Table 2:** Independent samples t tests for missing data approaches against reference value

Method	Mean	SD	*t*^a^	Degrees of Freedom	*P*	Cohen’s *d*
Reference	2.62	5.22	0	246	1	0
Complete cases	1.39	2.63	-2.09	176	0.04	-0.31
Mean imputation	1.39	1.73	-2.50	246	0.01	-0.35
LOCF	4.85	5.43	3.29	246	0.001	0.42
Regression imputation	1.39	2.37	-2.38	246	0.01	-0.32
EM imputation	3.09	3.85	0.809	246	0.42	0.10
NORM	3.14	9.55	0.534	246	0.53	0.07
MICE	3.06	4.30	0.730	246	0.47	0.09
SPSS 17 MI	1.49	2.03	-2.26	246	0.03	-0.31
Amelia II	2.88	3.33	0.468	246	0.64	0.06

^a^ Independent samples *t* tests

To supplement the visual analysis with statistics, Table 2 shows mean, standard errors, *t* statistics, and Cohen’s *d* effect sizes. The *t* and *d* values quantify the differences between the reference value and each of the imputed datasets. The lower its *t* statistic, the more the mean value obtained after application of a missing data approach resembles the reference value. This is an indication of the validity of an imputation method. To further indicate the extent to which the imputation results differ from the reference value, effect sizes were calculated using Cohen’s *d*. For this application, smaller effect sizes indicate better imputation results. The standard deviations for mean imputation, regression imputation, and SPSS multiple imputation are much smaller than the reference confidence interval. This could potentially lead to anticonservative testing results and, therefore, inflated (or increased) risk for type I error (false positives). NORM produced much larger confidence intervals; thus, NORM may lead to an increased risk for type II error (false negative).

**Figure 2:**
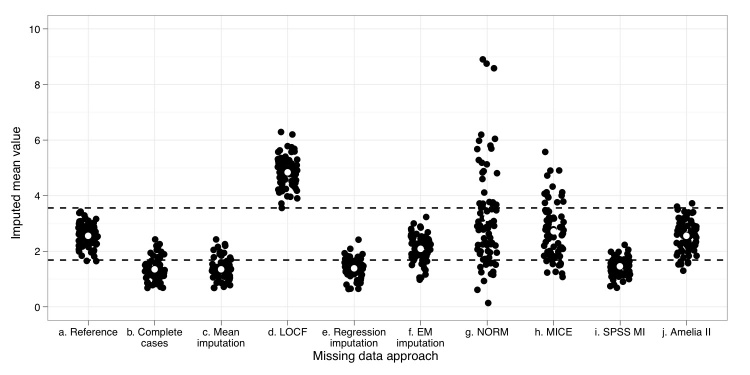
Repeated application of nine missing data approaches

Figure 2 provides insight in the reliability of the nine missing data approaches. The white dots in this figure show the arithmetic mean of the 75 bootstrapped samples. The area between the two dashed horizontal lines corresponds to the 95% confidence interval of the reference value. Each black dot indicates the arithmetic mean of one of the bootstrapped samples. As in Figure 1, some horizontal jitter was added to improve the visual presentation of the plotted data.

The nine approaches differed remarkably in the robustness and, therefore, in the reliability of their results. The largest difference between the simulated datasets was produced by the NORM software package, with some of the highest mean values being eight times larger than the smallest. The lowest variance was seen in the complete cases, mean imputation, regression imputation and SPSS MI. LOCF, EM imputation, MICE, and Amelia II showed an average amount of inter-dataset variance.

**Table 3:** Coverage of the reference confidence interval for imputed means

Missing Data Approach	Coverage Proportion	Variance of Bootstrapped Sample
Complete cases	0.15	0.088
Mean imputation	0.15	0.088
LOCF	0	0.381
EM imputation	0.83	0.206
Regression imputation	0.17	0.105
NORM	0.43	3.027
MICE	0.71	0.622
SPSS MI	0.23	0.093
Amelia II	0.96	0.205

Table 3 shows the variance of the means and the coverage for each approach. For an estimation to be maximally valid and reliable, at least 95% of the means obtained from the application of an approach should be within the confidence interval of the reference value [12]. The highest coverage was obtained by the application of Amelia II. This approach was actually the only one to reach the criterion of greater than 95% coverage. From the single imputation approaches, EM imputation yielded the highest coverage proportion.

## Discussion

In this paper, the application of nine approaches for handling missing data is presented and compared. The most valid result was obtained using multiple imputations from the Amelia II algorithm, closely followed by MICE, NORM, and EM imputation. However, due to the large standard errors resulting from the NORM algorithm, the power of the analysis based on this dataset was much lower than the power of an analysis using MICE or Amelia II would have been. The results obtained using the other tested approaches differed significantly from the reference value and can therefore be considered as less valid.

Although complete cases, mean imputation, regression imputation, and SPSS multiple imputation led to reliable results in the sense of small variance between the bootstrapped means (Figure 2), their application resulted in less valid parameter estimations (ie, the bootstrapped means are consistently lower than the reference mean) and their coverage was well below 95%. Optimal coverage was achieved using Amelia II, followed by EM imputation. Application of these two methods on the example dataset led to the most valid and reliable results. In general, it can be concluded that the more advanced approaches led to better results. Other authors have tested some of the presented approaches under both lower and higher missingness rates than the 50% in this study, with comparable results [ie, 12,25].

To mimic the real-life missing data problems more closely in this study, missingness was imposed on a variable containing count data (alcohol consumption counts). However, it should be noted that none of the presented approaches were specifically designed for the imputation of nonnormally distributed count data: specific missing data approaches for this type of data are currently lacking. From the Schafer suite, in addition to NORM, one could select CAT or MIX packages as an alternative, as these are intended for categorical or mixed datasets; however, these programs are also limited with regard to the imputation of missing count data. On the other hand, according to [12], excellent performance can be reached by imputing nonnormal variables under normality assumptions with no transformations. Based on the current study, it can be concluded that some methods can handle nonnormal count data well, while others perform less than optimally in such situations.

To evaluate the selected methods under more ideal conditions as well, the methods were retested using a normally distributed variable with missingness imposed under the same 50% MAR pattern (data not presented here). Differences between the methods became smaller; the less-than-optimal methods led to better results under these conditions. Multiple imputation still led to optimal results, and among the multiple imputation methods, the best results were reached using Amelia II.

Both EM imputation and Amelia II performed reasonably well in this study. EM imputation produces maximum likelihood estimates for the missing values, thus approaching true sample means and variances for an incomplete variable. However, being a single imputation method, the accuracy or inaccuracy of this estimation process is not accounted for in the variances of the resulting estimators. This leads to smaller variances, smaller confidence intervals, and therefore a greater risk of finding significant differences between variables when there are no actual differences (type I error, false positive). This shortcoming of EM imputation and other single imputation approaches marks the biggest advantage of multiple imputation. The latter captures uncertainty due to missingness of data in the variance between the generated datasets, making the estimators from multiple imputed datasets less prone to this type I error.

The main reason why MI is not used more often is probably due to the perceived complexity of its application. Working with more than one instance of the dataset may seem discouraging to researchers without extensive statistical knowledge or interest. Second, the fact that widely used statistical packages until recently did not natively support multiple imputation makes it understandable that most researchers using these software programs do not directly chose to apply this technique in case of missing data. In that sense, the introduction of multiple imputation in recent releases of statistical software (ie, the “mi” command in Stata 11 and the multiple imputation module in SPSS 17) may mark a leap forward. Positive experiences with the new “mi” command in Stata have been reported. However, under the conditions in the presented studies, the results obtained with SPSS 17 multiple imputation were less than optimal.

To conclude, this paper introduced both the implications and the practical use of data techniques to a wide, nonstatistical audience. Using the software packages tested and described in this paper, multiple imputation is feasible for any researcher in the eHealth field or related disciplines. The use of these approaches may invoke a considerable improvement of the validity of results obtained from datasets with missing values.

## Abbreviations

AIAR: Amsterdam Institute for Addiction Research
CONSORT: consolidated standards of reporting trials
EM: expectation maximization
LOCB: last observation carried backward
LOCF: last observation carried forward
MAR: missing at random
MCAR: missing completely at random
MI: multiple imputation
MNAR: missing not at random
RCT: randomized controlled trial
